# Lectotypification of *Plagiothecium mauiense*, a Hawaiian synonym of *Plagiothecium longisetum* (Plagiotheciaceae)

**DOI:** 10.3897/phytokeys.177.64042

**Published:** 2021-04-21

**Authors:** Grzegorz J. Wolski, Jarosław Proćków

**Affiliations:** 1 Department of Geobotany and Plant Ecology, Faculty of Biology and Environmental Protection, University of Lodz, ul. Banacha 12/16, 90-237 Lodz, Poland University of Lodz Lodz Poland; 2 Department of Plant Biology, Institute of Environmental Biology, Faculty of Biology and Animal Science, Wrocław University of Environmental and Life Sciences, ul. Kożuchowska 7a, 51-631 Wrocław, Poland Wrocław University of Environmental and Life Sciences Wrocław Poland

**Keywords:** Baldwin collection, Codes of Botanical Nomenclature, *Orthophyllum* section, typification

## Abstract

In 2020, *Plagiothecium mauiense* Broth. was recognised as a synonym of *P. longisetum* Lindb.; however, due to the inability to compare all known original material, the conducted taxonomic analysis was not completed with lectotypification of the name. Syntypes of *P. mauiense* were found in four American herbaria: Harvard University Herbarium (FH00220142), Miami University Herbarium (MU 000000546), New York Botanical Garden Herbarium (NY01256708) and Yale University Herbarium (YU 233890). Considering the condition of the found material and Articles 9.3, 9.11 and 9.12 of the International Code of Nomenclature for algae, fungi and plants (*Shenzhen Code*) that is currently in force, a specimen NY01256708 was proposed to be the lectotype of *P. mauiense*.

## Introduction

V.F. Brotherus described a new species, *Plagiothecium mauiense* (Brotherus 1927), based on specimens collected by D.D. Baldwin. A detailed comparative taxonomic analysis of the qualitative and quantitative features of the original material confirmed that the name is synonymous with *P. longisetum* Lindb. Additionally, the conducted research found all original specimens of *P. mauiense* to be syntypes (Wolski and Proćków 2020).

Unfortunately, to date, it was not possible to compare all known original material, so the lectotypification of *P. mauiense* was not completed (Wolski and Proćków 2020). However, this now appears possible and is the purpose of the present paper.

Wolski and Proćków (2020) confirmed that syntypes of *P. mauiense* are deposited in four American herbaria: Harvard University Herbarium (FH00220142), Miami University Herbarium (MU 000000546), New York Botanical Garden Herbarium (NY01256708) and Yale University Herbarium (YU 233890). Although it was possible to take on loan the described material from only two of them (Herbaria NY and YU), all four herbaria were able to provide the necessary assistance, as well as a wealth of data, including microscopic photographs of the described original material.

**Figure 1.**
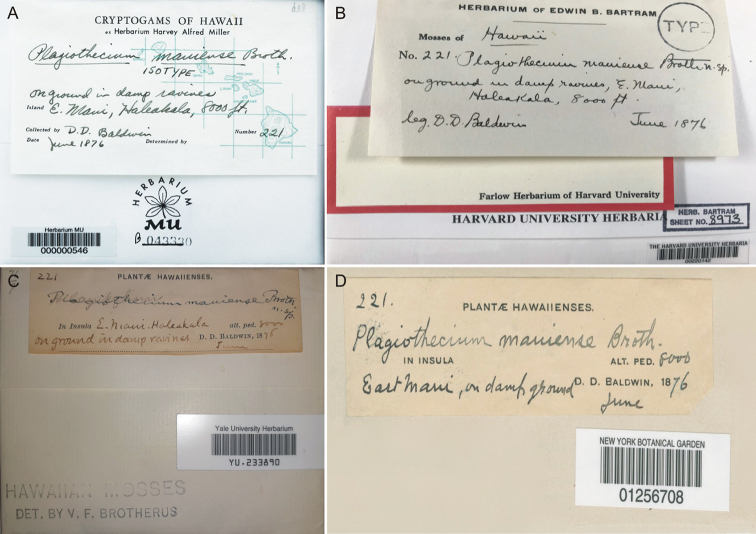
Envelope fronts of four syntypes of *Plagiothecium mauiense***A** MU 000000546 (photo from the Consortium of North American Bryophyte Herbaria, accessed 15 Nov 2020) **B** FH00220142 (photo by Genevieve E. Tocci) **C** YU 233890 **D** NY01256708. (**C, D** photo by Grzegorz J. Wolski).

The analysis of the obtained data showed that all syntypes on the envelopes are described identically, of course, apart from the details related to individual herbaria or additional annotations from persons who previously reviewed the material. The envelopes of the analysed syntypes give a name of the species with the abbreviation of the author’s name “*Plagiothecium mauiense* Broth.”; collection number “221”; collector’s name “D.D. Baldwin”; date “June 1876”; habitat “on ground in damp ravines”; location “E. Maui, Haleakala” and the relative height at which these plants grew “8000 ft.” (Fig. 1).

However, the analysis of the protologue indicates that the above data differ from what was published in 1927. Regarding the locality and habitat, after the description of the features of *P. mauiense* and before remarking that it is similar to “*P. silvatico* (Hudson)”, Brotherus (1927) provides: “Maui: on ground, elevation 2,400 meters (B. 221)”. This is obviously a much poorer description than that contained on the envelopes of all original specimens (Fig. 1). Nevertheless, all of them are syntypes of *P. mauiense* collected from Hawaii (i.e. specimens No. 221 from the D.D. Baldwin collection), on the basis of which V.F. Brotherus (1927) in *Hawaiian Mosses* described *P. mauiense*.

**Figure 2.**
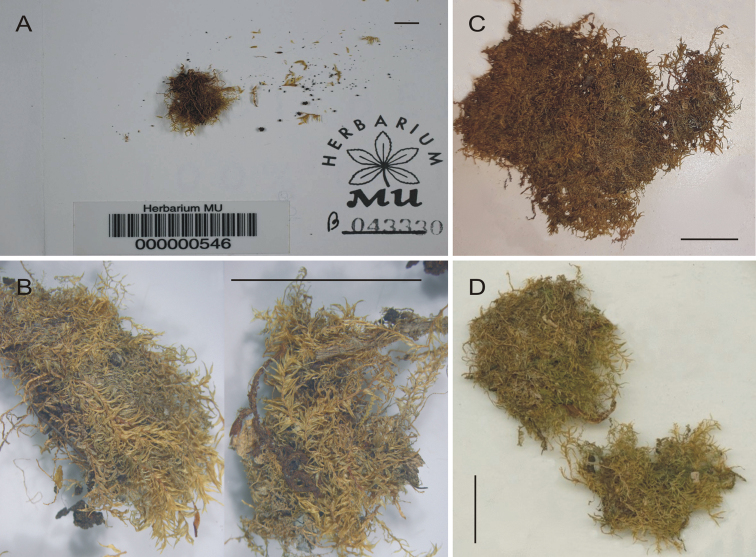
Syntypes of *Plagiothecium mauiense***A** MU 000000546 (photo by Mike Vincent) **B** FH00220142 (photo by Genevieve E. Tocci) **C** YU 233890 **D** NY01256708. (**C, D** photo by Grzegorz J. Wolski). Scale bar: 2 cm.

The analysis of the contents of the envelopes indicates that not all specimens are preserved in good condition. The specimen stored at the Miami University Herbarium (MU 000000546) is the smallest one within the analysed original material, with few loose stems (Fig. 2A). The specimen deposited in the Yale University Herbarium (YU 233890) is represented by a fairly large turf, but it is stuck to a small piece of paper. The use of glue could explain the change in colour of its turf: it is extremely dark compared to other specimens (Fig. 2C). The specimens stored at the Harvard University Herbarium (FH00220142) and New York Botanical Garden Herbarium (NY01256708) are represented by two quite large pieces of well-preserved turfs (Fig. 2B, D).

Taking into account the above data and the fact that the specimen stored at NY is well preserved and has recently been described in detail (Wolski and Proćków 2020), as well as the fact that this material (NY01256708) consists of an additional permanent preparation of a large fragment of the stem, that was probably made by Iwatsuki in 1970 and, according to Article 9.3 of the *Shenzhen Code* (Turland et al. 2018), stating that “A lectotype is one specimen or illustration designated from the original material (…) as the nomenclatural type, in conformity with Art. 9.11 and 9.12, if the name was published without a holotype (…)” and that “in lectotype designation, an isotype must be chosen if such exists, or otherwise a syntype or isosyntype if such exists” (Art. 9.12), we propose that specimen NY01256708 should be designated as the lectotype of *P. mauiense* Broth. (Fig. 2D).

Additionally, due to the fact that two specimens were not physically available to us, we will send a request to the Harvard University Herbarium (FH00220142) and Miami University Herbarium (MU 000000546) to change the status of these specimens to isolectotypes, labelling them appropriately, after the article is published.

## Taxonomic treatment

### *Plagiothecium longisetum* Lindb., Contributio ad Floram Cryptogamam Asiae Boreali-Orientalis, Acta Soc. Sci. Fenn. 10: 232 (1872)

Type. [Japan], ad Nikosan ins. Kiusiu, [fertile], 16 Junii 1863, *S.O. Lindberg s.n.* (lecto-: H-SOL 1563 011!, isolecto-: PC0132572!, S-B160017) = *P. mauiense* Broth., Bernice P. Bishop Museum Bulletin 40: 28 (1927). – ***Lectotype*** (designated here): [United States], Hawaii, E Maui, Haleakala, 8000 ft., in damp ravines, fertile, June 1876, *D.D. Baldwin 221* (NY01256708!); isolecto-: FH00220142 (available online)!, MU000000546 (available online)!, YU233890!).

Thus, the selection of the lectotype formally completed the taxonomic revision of the original material of *Plagiothecium mauiense*, collected by D.D. Baldwin from Hawaii, U.S.A.
